# Selections

**Published:** 1894-03

**Authors:** 


### Selections.

### CREOSOTAL.

This is the name which Professor Brisonnet, of the School of Medicine, Tours, gives to the body obtained by combination of carbonic acid and creosote (*Répert. de Pharm.*). The product is likely to be of considerable therapeutic value, for it is a neutral, bland, sweet, oily liquid, without odor, non-irritating to the mucous membrane, and is readily borne by the stomach. It is made by acting upon sodium-creosote with chloro-carbonic acid, $COCl_2$, in alkaline solution. The creosotal separates and sinks to the bottom of the mixture. It is collected, washed with a weak cold solution of alkali, and any adhering water is driven off by a gentle heat. The specific gravity of the purified creosotal is 1.165; it is insoluble in water, glycerin, and dilute alcohol, but soluble in all proportions of strong alcohol, ether, chloroform, and benzin. A hundred parts of it are equal to ninety of creosote, yet it has been given in doses of ten, fifteen, and twenty grammes a day without disturbing digestion. In the intestines it is resolved into its components, creosote and carbonic acid, and creosote is found in the urine half an hour after a dose has been taken. Its use is indicated in tuberculosis and other diseases for which creosote is prescribed.—*Chemist and Druggist*, February 18, 1893.

### THE ANTIDOTE FOR ARSENIC.

Dr. Squibb recommends the following as a simple method of preparing hydrated oxide of iron, the antidote for arsenic, one of its chief advantages being that the ingredients are always easily obtained: Take of tinctura ferri chloridi, four ounces; aqua fortis, four ounces; mix in a vessel of twelve ounces capacity, and add aqua ammoniæ, one drachm. Shake well, pour it on a large wet muslin drainer, wring out the water and alcohol, and wash with fresh water. The stomach having been evacuated by emetics while the antidote was being prepared, give four fluidounces at once, to be followed by an emetic. Then give two ounces every ten minutes.—*N. Y. Med. Times.*

### THIOCAMF.

This name was given by Professor Emerson Reynolds to a liquid devised by him as a disinfectant, and described in a paper read by him at a meeting of the Royal Dublin Society four years ago. It is made by bringing sulphur-dioxide gas into contact with camphor and dissolving in the resulting liquid various antiseptics, among them phellandrene and benzoic acid. This liquid can be kept in bottles without pressure, but on exposing it to the air in a thin layer it gives off "relatively enormous volumes" of sulphur-dioxide gas. Reflecting upon this property possessed by the liquid, Dr. George F. Duffey conceived the idea of using it as an intestinal antiseptic. He gives an account of his use of thiocamf as a remedy in the May number of the *Dublin Journal of Medical Science.*

As regards the external use of thiocamf, Dr. Duffey's experience includes two cases of scabies. One was very severe and complicated with extensive eczema. A four-per-cent. solution of the drug in olive oil was applied, and the patient was cured in fourteen days. In the other case, in which there was but little eczema, a cure was effected in five days. From the effects of the pharmacopœial solution of sulphurous acid in parasitic skin-diseases he thinks that an oily solution of thiocamf would probably be of use in pityriasis versicolor, favus, and other dermatophytic affections. In the case of a paralyzed woman who had a large bedsore of the sacral and gluteal regions, gangrenous and emitting a most offensive odor, applications of an oily solution—four per-cent. at first, then six-per-cent.—"quickly removed the fetor, diminished the discharge, and caused the sore, after the removal of the slough, to assume a clean, healthy appearance." A four-per-cent. solution came to be largely used in the out-patient service of the City of Dublin Hospital, in which institution Dr. Duffey's trials of thiocamf were made, for dressing ulcers and wounds, and was found very efficient in keeping the parts free from fetor and in checking the discharge. The application of such a solution to a raw surface causes a slight and evanescent sensation of heat and prickling; if a stimulating effect is required, the strength of the solution may be increased.—*N. Y. Med. Journ.*

