# Antidote for Arsenic

**Published:** 1894-05

**Authors:** Edward C. Kirk

**Affiliations:** Philadelphia


### Domestic Correspondence.

### ANTIDOTE FOR ARSENIC. CORRECTION OF FORMULA.

PHILADELPHIA, March 17, 1894.

TO THE EDITOR:

SIR,—At page 207 of the March issue of your valuable journal appears a clipping from the *New York Medical Times* of June, 1893, entitled "The Antidote for Arsenic." The prescription as given contains an error which may cause damage to life if allowed to pass uncorrected, and is as follows: "Take of tinctura ferri chloridi, four ounces; *aqua fortis, four ounces;* mix in a vessel of twelve ounces capacity, and add aqua ammoniæ, one drachm," etc. The object of the mixture is to produce the hydrated oxide of iron in the moist state, which is the recognized chemical antidote for arsenical poisoning. It will be readily seen, however, that the addition of an equal amount of aqua fortis to the tinctura ferri chloridi would not of itself produce a precipitate of ferric oxide, and would also effectually prevent its precipitation by the one drachm of ammonia to be subsequently added. The resulting mixture made according to the formula given would consist of approximately equal parts of tinctura ferri chloridi and strong nitric acid with some ammonia nitrate. Inasmuch as the directions are, "after pouring the mixture on a large wet muslin drainer, to wring out the water and alcohol, wash with fresh water, etc., give four *fluidounces* at once, and after an emetic to give two fluidounces every ten minutes," it would seem highly probable that one not familiar with the chemistry or pharmacology of the subject might presume that the filtrate was intended to be taken, especially as no precipitate would be formed under the circumstances; in fact, no reference to the formation of a precipitate is made in the article.

The direction to add aqua fortis is no doubt an accidental error, due to careless proof-reading of the original article in the *New York Medical Times*, in which journal, page 94 of the June issue of last year, it is printed "aqua fort.," and is almost certainly intended for *aqua font.*, or ordinary hydrant water. Dr. Squibb states that he was not the author of the communication, and the *New York Medical Times* people are silent upon it after specific inquiry as to their source of information. Squibb's antidote for arsenical poisoning consists of a diluted solution of tersulphate of iron and a mixture of magnesia and water (1 to 10) dispensed in separate packages which, when mixed together, make the official ferri oxidum hydratum cum magnesia ready for use. I have taken the liberty of calling attention to this error, which is a serious one in that the prescription as published is not in any respect an antidote for arsenic, but if swallowed in the amounts directed would be certainly and rapidly fatal.

Yours truly,

EDWARD C. KIRK.

